# *Bartonella* spp. Infections, Thailand

**DOI:** 10.3201/eid1604.090699

**Published:** 2010-04

**Authors:** Saithip Bhengsri, Henry C. Baggett, Leonard F. Peruski, Christina Morway, Ying Bai, Tamara L. Fisk, Anussorn Sitdhirasdr, Susan A. Maloney, Scott F. Dowell, Michael Kosoy

**Affiliations:** Thailand Minstry of Public Health–US Centers for Disease Control and Prevention Collaboration, Nonthaburi, Thailand (S. Bhengsri, H.C. Baggett, L.F. Peruski Jr, T.L. Fisk, S.A. Maloney); Thailand Ministry of Public Health, Nonthaburi (A. Sitdhirasdr); Centers for Disease Control and Prevention, Atlanta, Georgia, USA (S.F. Dowell); Centers for Disease Control and Prevention, Fort Collins, Colorado, USA (C. Morway, Y. Bai, M. Kosoy).

**Keywords:** Bartonella, bacteria, Thailand, letter

**To the Editor:** *Bartonella* are fastidious hemotropic gram-negative bacteria with a worldwide distribution. In Thailand, *Bartonella* species have been demonstrated in mammalian hosts, including rodents, cats and dogs, and in potential vectors, including fleas (*1*–*4*). However, data on human infection have been limited to case reports (*5*,*6*) and 1 seroprevalence survey, which found a 5.5% prevalence of past *B. henselae* infection (*7*). No studies have systematically assessed the frequency, clinical characteristics, or epidemiology of human *Bartonella* infections in Thailand.

We conducted a prospective study to determine causes of acute febrile illness in 4 community hospitals, 2 in Chiang Rai (northern Thailand) and 2 in Khon Kaen (northeastern Thailand). We enrolled patients >7 years of age with a temperature >38°C who were brought to study hospitals for treatment from February 4, 2002, through March 28, 2003. Patients were excluded if they had a history of fever for >2 weeks or an infection that could be diagnosed clinically. Acute-phase serum samples were collected at the time of enrollment and convalescent-phase serum samples 3–5 weeks later. We enrolled nonfebrile control patients >14 years of age who had noninfectious conditions; acute-phase serum samples were collected. Clinical information was abstracted from patient charts. Nurses conducted physical examinations and personal interviews to collect information on patients’ demographic characteristics, exposures to animals, and outdoor activities.

Serum samples were tested for immunoglobulin (Ig) G antibodies to *Bartonella* spp. by immunofluorescent antibody assay at the Bartonella Laboratory of the Centers for Disease Control and Prevention, Fort Collins, CO, USA. Strains used for antigen production were: *B. elizabethae* (F9251), *B. henselae* (Houston-1), *B. quintana* (Fuller), and *B. vinsonii* subsp. *vinsonii* (Baker). Homologous hyperimmune serum specimens were produced in BALB/c mice as previously described (*8*). *Bartonella* infection was considered confirmed in febrile patients who had a >4-fold rise in IgG antibody titers and a convalescent-phase titer >64. Probable infection was defined as 1) a 4-fold antibody titer rise but convalescent-phase titers of 64, or 2) high and stable titers (>512 in acute-phase and convalescent-phase serum samples), or 3) acute-phase titer >512 with a >4-fold titer fall. Paired serum samples from febrile patients were also tested for serologic evidence of other common causes of febrile illness in Southeast Asia.

Febrile patients with acute-phase and convalescent-phase IgG antibody titers <128 were considered not to have *Bartonella* infection; we compared demographic and clinical characteristics of these patients to *Bartonella*-infected patients. To evaluate potential risk factors, we compared *Bartonella*-infected case-patients >14 years of age without serologic evidence of other infections (n = 20) to nonfebrile controls with IgG to *Bartonella* <128 (n = 70). Age adjusted odds ratios (AORs) with 95% confidence intervals (CIs) were calculated.

Serologic testing was completed on paired serum samples for 336 (46%) of 732 febrile patients enrolled; 92 (27%) had serologically confirmed (50) or probable (42) *Bartonella* infections. Thirty-five (38%) of these 92 had serologic evidence of infection with another pathogen. The remaining 57 *Bartonella*-infected case-patients (34 confirmed, 23 probable) had a median age of 19 years (range 7–72 years); 65% were males, 47% were students, and 35% were rice farmers. Common clinical characteristics of *Bartonella*-infected patients included myalgias (83%), chills (79%), and headache (77%). Thirty (60%) patients had anemia (hemoglobin level <13 mg/dL); 18 (32%) had a hemoglobin level <12 mg/dL, and 4 (7%) had <11 mg/dL. When compared with 193 febrile patients without *Bartonella* infection, the 57 *Bartonella*-infected patients were similar in age and sex but were more likely to be rice farmers and were more likely to have leukocytosis (Table). Compared with the 70 nonfebrile controls, *Bartonella-*infected case-patients were more likely to report tick exposure (32% vs. 7.9%; AOR = 5.6, 95% CI 1.5–21) and outdoor activities (55% vs. 31%; AOR = 2.7, 95% CI 1.0–7.4) during the 2 weeks before illness onset. Prevalence of reported rat exposure and animal ownership (cats, dogs, pigs, cows, or buffaloes) was similar among case-patients and controls.

**Table:** Demographic and clinical characteristics of febrile patients with *Bartonella* infection compared with febrile patients who had no evidence of *Bartonella* infection, Thailand, 2002–2003*

Variables	No. (%) *Bartonella*-infected case-patients,† n = 57	No. (%) non–case-patients,‡ n = 193	p value
Median age, y (range)	19 (7–72)	20 (7–79)	0.9
Male sex	37 (64.9)	113 (58.5)	0.4
Occupation			
Student	27 (47.4)	84 (43.5)	0.6
Rice farmer	20 (35.1)	40 (20.7)	0.03
Business	3 (5.3)	13 (6.7)	0.7
Other§	7 (12.3)	56 (29.0)	0.01
Signs and symptoms			
Headache	44 (77.2)	161 (83.4)	0.3
Eye pain	17 (29.8)	58 (30.1)	1.0
Myalgias	47 (82.5)	141 (73.1)	0.1
Extremity pain	39 (68.4)	115 (59.6)	0.2
Joint pain	26 (45.6)	74 (38.3)	0.3
Vomiting	22 (38.6)	80 (41.5)	0.7
Abdominal pain	12 (21.1)	64 (33.2)	0.08
Rash	7 (12.3)	17 (8.8)	0.6
Lymphadenopathy	9 (15.8)	24 (12.4)	0.5
Laboratory results			
Anemia (Hb <13 mg/dL)	30 (60.0)	93 (50.3)	0.2
Thrombocyte count <100,000/mm^3^	5 (8.9)	14 (7.4)	0.7
Leukopenia (leukocytes <5,000/mm^3^)	10 (17.5)	63 (32.6)	0.03
Leukocytosis (leukocytes >11,000/mm^3^)	20 (35.1)	33 (17.1)	<0.01
Creatinine >1.5 mg/dL	5 (8.9)	12 (6.2)	0.5
Bilirubin >1.3 mg/dL	6 (10.7)	19 (9.8)	0.8
Alkaline phosphatase >121 IU/L	36 (64.3)	132 (68.8)	0.5
AST >36 IU/dL	18 (32.1)	88 (45.6)	0.07
ALT >36 IU/dL	9 (16.1)	50 (25.9)	0.1

*Hb, hemoglobin; AST, aspartate aminotransferase; ALT, alanine aminotransferase. †Excluded *Bartonella*-infected patients with serologic evidence of infection with other common pathogens (dengue virus, *Leptospira*, or *Burkholderia pseudomallei*; n = 35). ‡Febrile patients with acute-phase and convalescent-phase immunoglobulin G titer against *Bartonella* species <128. §Other occupation includes housewife, government officer, day laborer, or construction worker.

We describe the frequency and clinical characteristics of acute *Bartonella* infection among febrile patients in Thailand. Over 25% of patients with undifferentiated febrile illness had serologic evidence of *Bartonella* infection (including 15% serologically confirmed). Our findings indicate that *Bartonella* infections may be common and underrecognized causes of acute febrile illness in rural Thailand. Although our results are limited by lack of culture confirmation, we used conservative case definitions for serologic diagnosis and therefore believe that most cases represent true *Bartonella* infections. The common clinical features of anemia and leukocytosis and the frequent tick exposure and outdoor activity are consistent with known features of *Bartonella* infections and lend support to serologic findings. Because of the potential for serologic cross-reactivity between *Bartonella* species, we did not attempt species identification. The case-control study was therefore limited by grouping case-patients that were likely infected with different *Bartonella* species for which risk factors may differ. Such studies could lead to meaningful recommendations for prevention and control of *Bartonella* infections. Additional epidemiologic and transmission studies are needed to improve understanding of risk factors, identify key animal reservoirs and vectors, and ascertain transmission dynamics.
